# Longitudinal data of multimorbidity and polypharmacy in older adults in Taiwan from 2000 to 2013

**DOI:** 10.37796/2211-8039.1013

**Published:** 2020-06-05

**Authors:** Shih-Wei Lai, Kuan-Fu Liao, Cheng-Li Lin, Cheng-Chieh Lin, Chih-Hsueh Lin

**Affiliations:** 1 College of Medicine, China Medical University, Taichung, Taiwan; 2 Department of Family Medicine, China Medical University Hospital, Taichung, Taiwan; 3 College of Medicine, Tzu Chi University, Hualien, Taiwan; 4 Division of Hepatogastroenterology, Department of Internal Medicine, Taichung Tzu Chi Hospital, Taichung, Taiwan; 5 Management Office for Health Data, China Medical University Hospital, Taichung, Taiwan

**Keywords:** multimorbidity, older adults, polypharmacy

## Abstract

**Objective:**

The objective of the study was to evaluate the prevalences and trends of multimorbidity and polypharmacy in older adults in Taiwan.

**Methods:**

An observational study was performed using the 2000-2013 database of the Taiwan National Health Insurance Program (analysis in 2018). Participants ≥65 years were included in the study. Multimorbidity was defined as participants having two or more chronic diseases annually. Polypharmacy was defined as the average daily number of prescribed medications ≥5.

**Results:**

The prevalences of multimorbidity were 42.4% in 2000 and 56% in 2013. The prevalences of polypharmacy were 22.9% in 2000 and 32.1% in 2013.

**Conclusions:**

From 2000 to 2013, multimorbidity and polypharmacy were prevalent among older adults in Taiwan. Public health efforts to intervene the primary prevention for chronic diseases should be considered in older adults.

## 1. Introduction

Multimorbidity and polypharmacy in older adults are major public concerns worldwide. Previous studies only focused on the prevalences of multimorbidity and polypharmacy of the year studied. For example, Kirchberger et al reported that the prevalence of multimorbidity (≥2 diseases) was 58.6% in a cross-sectional study of 4127 persons aged 65–94 years in 2012.[1] Junius-Walker et al reported that the prevalence of polypharmacy (≥5 prescribed medications) was 26.7% in a cross-sectional study of 466 older adults in 2007.[2] Moreover, only longitudinal data can well illustrate the long-term trends of multimorbidity and polypharmacy in older adults. Similarly, the increasing number of aging population is also a global challenge. We make a rational hypothesis that there could be an interaction between aging population, multimorbidity, and polypharmacy in older adults. To evaluate this issue, we conducted an observational study to examine the 2000-2013 database of the Taiwan National Health Insurance Program.

## 2. Methods

### 2.1. Study design and data source

A population-based observational study was conducted to analyze the 2000-2013 database of the Taiwan National Health Insurance Program. The program was launched in March 1995 and has covered 99.7% of 23 million persons living in Taiwan. [3, 4] The number of people ≥65 years can be found in the Ministry of the Interior in Taiwan.[5].

### 2.2. Sampled participants

**Fig. 1:**
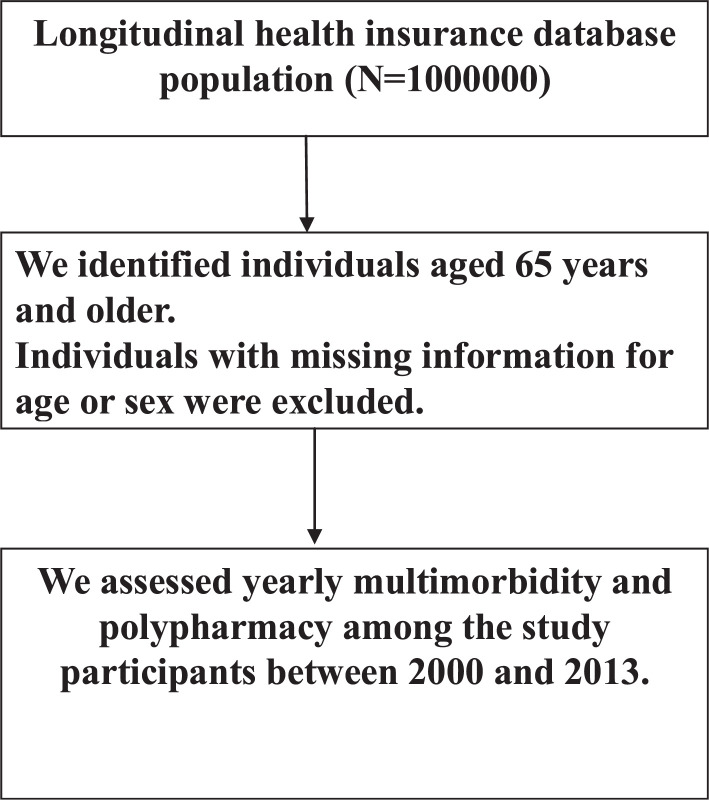
The study flowchart.

Participants ≥65 years were included in the study. Multimorbidity was defined as participants having two or more chronic diseases annually.[6] Chronic diseases included 98 diseases approved by the Ministry of Health and Welfare in Taiwan.[7] Medications studied were those medications prescribed for either chronic or acute conditions. Polypharmacy was defined as the average daily number of prescribed medications ≥5.[8-11] Fig. 1 disclosed the study flowchart.

### 2.3. Statistical analysis

We analyzed the number of chronic diseases (0, 1, 2-4, 5-9, and ≥10 diseases) and the number of prescribed medications (0, 1-4, 5-9, and ≥10 medications) per year from 2000 to 2013. The percentages were reported using these data. The aging population, multimorbidity, and polypharmacy were measured annually between 2000 and 2013 and examined with simple correlation regression. All analyses were performed using the SAS statistical software (version 9.2; SAS Institute, Inc., Cary, NC, USA).

## 3. Results

In Table 1, the prevalences of multimorbidity were 42.4% in 2000 and 56% in 2013. The prevalences of polypharmacy were 22.9% in 2000 and 32.1% in 2013. The prevalences of adults ≥65 years were 8.6% in 2000 and 11.5% in 2013 (*P* for trend <0.001).

**Fig. 2:**
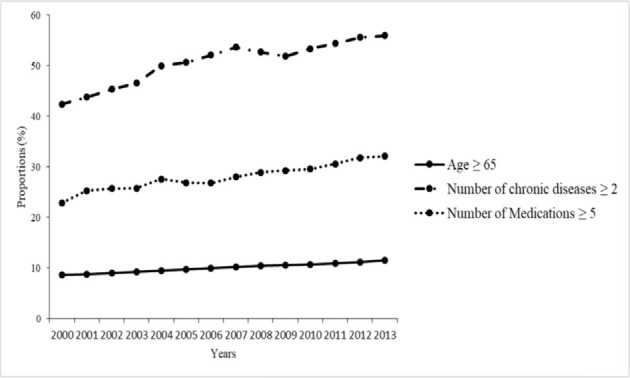
Long-term trends of aging population, multimorbidity, and polypharmacy in older adults in Taiwan from 2000 to 2013 (P for trend <0.001).

Fig. 2 disclosed that the prevalences of adults *≥* 65 years, multimorbidity, and polypharmacy increased at a substantial rate from 2000 to 2013. There seemed to be a parallel increase in the prevalences of aging population, multimorbidity, and polypharmacy in older adults from 2000 to 2013 (*P* for trend <0.001).

## 4. Discussion

In this population-based observational study, the prevalences of aging population, multimorbidity, and polypharmacy increased at a substantial rate from 2000 to 2013. There seemed to be a relationship of parallel increase between these three conditions. That is, older adults are at risk for multimorbidity and polypharmacy. Although older adults who have chronic diseases can get benefits from the effective medications but these medications also place those older adults at risk of getting harms. Polypharmacy in older adults is associated with an increased risk of falls, adverse drug reactions, potentially inappropriate medications, frailty, and others.[12-19] An observational using the same Taiwan database disclosed that the prevalence of use of 5 to 9 prescribed drugs daily was 26.3% and the prevalence of use of ≥10 prescribed drugs daily was 5.8% in older people in 2013.[20] An observational study in South Korea disclosed that the prevalence of polypharmacy (≥6 drugs) was 86.4% and the prevalence of major polypharmacy (≥11 drugs) was 44.9% in older people from 2010 and 2011.[21] Moreover, a cross-sectional study in Belgium disclosed that use of 5 to 9 drugs or ≥10 drugs was associated with an increased risk of potentially inappropriate medications (relative risk 2.27 and 4.04, respectively).[16] These results suggest that older adults in Taiwan who had polypharmacy might be potentially at risk of potentially inappropriate medications. In addition, one cohort study in Netherlands disclosed that multimorbidity or polypharmacy at baseline was associated with increased risk of mortality in people with intellectual disability (hazard ratio = 2.61,95% CI = 1.86-3.66; hazard ratio = 2.32, 95% CI = 1.70-3.16, respectively).[22] Hence, how to break such a relationship between aging population, multimorbidity, and polypharmacy is a public health problem.

The worldwide burden of aging population is increasing annually and currently there is no slowing trend. Polypharmacy is ubiquitous in older adults, and even though an abundance of intervention strategies, it remains uncontrolled. If older adults do not get sick, there might be a chance to break such a relationship between aging population, multimorbidity, and polypharmacy. From the point of preventive medicine, only primary prevention can make sense. Strategies for health promotion and specific protection should be intervened to keep older adults from getting chronic diseases. If chronic diseases can be prevented, the number of medications for treating chronic diseases can be reduced and then polypharmacy can be further reduced. Therefore, the relationship between aging population, multimorbidity, and polypharmacy can be broken.

Some limitations should be discussed. First, numerical definitions of polypharmacy do not always account for specific comorbidities present and make it difficult to assess safety and appropriateness of medications in the clinical setting. How to make a practical definition on polypharmacy is a future research direction. Second, Due to the limitation of the database used, the impact of “doctor shopping” on polypharmacy cannot be assessed. It indicates a future research direction.

We conclude that from 2000 to 2013, the prevalences of aging population, multimorbidity, and polypharmacy substantially increased in older adults in Taiwan. Public health efforts to intervene the primary prevention for chronic diseases should be considered in older adults.

## Supplementary materials



## Figures and Tables

**Table 1:** Prevalences (%) of aging population, multimorbidity, and polypharmacy in older adults in Taiwan from 2000 to 2013.

	2000	2001	2002	2003	2004	2005	2006	2007	2008	2009	2010	2011	2012	2013	*P* for trend
Age > 65	8.6	8.8	9.0	9.2	9.5	9.7	10	10.2	10.4	10.6	10.7	10.9	11.2	11.5	<0.001
Number of chronic diseases ≥ 2	42.4	43.8	45.4	46.6	50	50.7	52.1	53.7	52.7	51.9	53.4	54.4	55.6	56	<0.001
Number of Medications ≥ 5	22.9	25.3	25.7	25.8	27.6	26.9	26.8	28	28.9	29.3	29.6	30.6	31.8	32.1	<0.001
