# Supplementary material for: Longitudinal data of multimorbidity and polypharmacy in older adults in Taiwan from 2000 to 2013
Source: Biomedicine (Taipei). 2020 Jun 5;10(2):1–4. doi: 10.37796/2211-8039.1013 (PMC7608846; doi:10.37796/2211-8039.1013)
Supplement: Supplementary file 1 [file bmed-10-02-001-s001.doc]

**Longitudinal health insurance database population (N=1000000)**

**We identified individuals aged 65 years and older.**

**Individuals with missing information for age or sex were excluded.**

**We assessed yearly multimorbidity and polypharmacy among the study participants between 2000 and 2013.**
